# Artificial Intelligence Design for Race-Based Prostate Cancer Stage Classification With Multilayer Perceptron: Feature Selection Optimization Approach

**DOI:** 10.2196/82587

**Published:** 2026-04-16

**Authors:** Adithama Mulia, David Agustriawan, Marlinda Overbeek, Moeljono Widjaja, Vincent Kurniawan, Jheno Syechlo, Muhammad Imran Ahmad, Srinivasulu Yerukala Sathipati, Nilubon Kurubanjerdjit

**Affiliations:** 1 Department of Informatics, Faculty of Engineering and Informatics, Universitas Multimedia Nusantara, A Bldg, 5th Fl, Tangerang, 15810, Indonesia, 62 87781535936; 2 Faculty of Intelligent Computing, Universiti Malaysia Perlis, Kampus Pauh Putra, Perlis, Malaysia; 3 Center for Precision Medicine Research, Marshfield Clinic Research Institute, Marshfield, WI, United States; 4 School of Applied Digital Technology, Mae Fah Luang University, Chiang Rai, Thailand

**Keywords:** prostate cancer, multilayer perceptron, feature selection, DNA methylation, differentially methylated positions, race-aware model, explainable artificial intelligence

## Abstract

**Background:**

Prostate cancer progression exhibits significant variability influenced by biological and racial factors. DNA methylation profiling has shown potential in early cancer detection, but its integration with machine learning across racially diverse populations remains limited.

**Objective:**

This study aimed to develop a prostate cancer stage classifier for the majority White cohort using DNA methylation data and a multilayer perceptron (MLP) model in order to classify prostate cancer stages into early (stages I-II) and late (stages III-IV) stages and assess its performance when applied to other racial groups to highlight the need for race-specific models.

**Methods:**

Methylation and phenotype data from the TCGA-PRAD (The Cancer Genome Atlas Prostate Adenocarcinoma) dataset were processed using differentially methylated position (DMP) analysis to identify CpG sites correlated with cancer stages. These features were further refined through recursive feature elimination (RFE) and used to train MLP models. Shapley Additive Explanations (SHAP) and Local Interpretable Model-Agnostic Explanations (LIME) were used to interpret the model and identify key DNA methylation features contributing to model predictions.

**Results:**

The best-performing model achieved 95% accuracy and up to 99% area under the curve on the majority race (White) training data using 90 selected features. However, performance declined sharply in racial minority groups, revealing the effects of sample imbalance and race-specific methylation patterns. Feature importance examination indicated strong patterns within certain CpG sites driving model predictions.

**Conclusions:**

We propose a race-aware MLP model for prostate cancer stage classification using DNA methylation data, which has been optimized through DMP and RFE-based feature selection. SHAP and LIME confirmed the predictive relevance of selected CpG sites, supporting model transparency. The results highlight high performance within the White cohort but reveal poor generalization to racial minority groups, emphasizing the importance of race-specific modeling strategies.

## Introduction

Prostate cancer is one of the most frequently diagnosed cancers in men and a major cause of cancer-related deaths in the United States [1-3]. It is particularly prevalent among older individuals, with risk factors including age, family history, and lifestyle [4]. Biologically, prostate cancer is a heterogeneous disease, marked by distinct subtypes and gene expression profiles that influence prognosis and treatment outcomes [5-7]. These biological variants range in severity from slow-growing forms to aggressive and rapidly spreading types, affecting how the disease progresses and responds to therapy. This complexity poses a challenge for achieving accurate and consistent diagnosis across diverse patient populations.

The classification of prostate cancer typically depends on the Gleason score and androgen receptor status, which inform tumor aggressiveness and therapeutic response [8]. Genetic mutations, including those involving *TMPRSS2*-*ERG*, *PTEN*, and *SPOP*, have also been implicated in disease progression [9-11]. Advances in sequencing and molecular profiling have enabled the discovery of new biomarkers for targeted treatment [12], yet many classification models still fail to capture the nuanced biological variation across cancer stages.

Next-generation sequencing (NGS) has seen rapid technological advancements over the years and has become central to exploring epigenetic regulation in cancer [13]. DNA methylation, an epigenetic mechanism that modifies gene expression without altering the sequence, is key to identifying biomarkers and understanding cancer development. NGS enables high-throughput, genome-wide methylation profiling, facilitating the discovery of fine-grained methylation patterns and their association with specific mutations [14-16]. However, the vast complexity of methylation data demands computational approaches that can extract clinically actionable insights, which remains a key challenge.

Methylation data analysis, especially differentially methylated position (DMP) analysis, addresses the high dimensionality of methylation profiles by identifying individual CpG sites with significant methylation differences between groups [17]. DMP analysis offers single-site resolution and is better suited for classification tasks compared with region-based methods like differentially methylated regions, especially when individual CpG sites are used as input features [18]. Statistical methods, such as *t* tests and linear models, help quantify these differences [19]. Despite their advantages, DMPs are rarely used in conjunction with machine learning models like multilayer perceptron (MLP) for stage-specific cancer classification, highlighting a gap that this research aims to address. Beyond genetic variation, accumulating evidence shows that DNA methylation profiles differ substantially between populations and racial or ethnic groups, reflecting both ancestry and the environment. Genome-wide studies have reported population-specific methylation patterns at thousands of CpG sites, with over one-third of genes showing differential methylation between African and European populations, often driven by differences in allele frequencies and gene-environment interactions. Ethnic differences in methylation have been replicated in multiple cohorts and tissues, including the blood and brain, and frequently map to loci linked to metabolic traits and other complex diseases. This indicates that epigenetic regulation is not universally shared across racial or ancestral groups but is shaped by socially patterned exposures, lifestyle, and genetic background [20-22].

MLP is a neural network model widely used for pattern recognition and classification in fields like bioinformatics, particularly cancer diagnostics [23-26]. It can effectively manage high-dimensional data, such as DNA methylation data, outperforming many traditional statistical approaches. MLP has been shown to identify cancer-specific biomarkers and classify cancer stages with high accuracy [27-29]. Beyond that, MLP also supports personalized therapy by improving diagnostic consistency. However, no prior studies have combined DMP-based feature selection with MLP for stage-specific cancer classification, making this approach a novel contribution to the field. This framework provides accurate race-based classification of prostate cancer for diagnosed individuals, offering an effective tool for rapid initial screening and preoperative assessment.

Although machine learning has significantly advanced cancer classification, current genome-based models often neglect racial disparities, particularly in DNA methylation patterns. A study by Abdollahi et al [30] introduced a radiomic-based framework for prostate cancer evaluation, utilizing machine learning techniques and magnetic resonance imaging (MRI) features to predict the treatment response, Gleason score, and cancer stage. The study involved 33 patients with prostate cancer who underwent T2-weighted (T2W) and apparent diffusion coefficient (ADC) MRI scans before and after intensity-modulated radiation therapy. Radiomic features were extracted from both image types, and univariate analysis combined with paired *t* tests identified significant features associated with treatment response. Feature selection and classification were performed using 10-fold cross-validation across various image sets, and the predictive performance of post-T2W radiomic models was the highest (area under the curve [AUC]=0.632), followed by pre-ADC models (AUC=0.626) and pre-T2W models (AUC=0.610). Additionally, T2W-based models had a mean AUC of 0.739 for Gleason score prediction, while ADC-based models performed better in stage prediction, with a mean AUC of 0.675. A study by Hartenstein et al [31] evaluated 3 convolutional neural networks (CNNs) trained to detect lymph node infiltration by prostate cancer, using contrast-enhanced computed tomography images. The models were tested against expert radiologists and achieved comparable performance. The best-performing CNN, trained on a status-balanced dataset, reached a high AUC of 0.90, primarily by learning anatomical context. In contrast, a location-balanced CNN and a segmentation-masked (xMask) CNN reached lower AUCs of 0.858 and 0.677, respectively. Random forest classifiers using only nodal volume and location performed well on status-balanced data (AUC=0.90) but poorly on location-balanced data (AUC=0.677), reinforcing that CNNs leveraged anatomical features in classification. A similar study by Eissa et al [32] utilized DNA methylation profiles to investigate the impact of feature selection in reducing the dimensionality of methylation data through metaheuristic techniques. The study was structured in 2 stages: the first stage focused on feature selection, and the second stage focused on developing a deep neural network (DNN) model to classify samples based on malignancy status and cancer type. The proposed method achieved competitive results compared with existing approaches, with strong performance in terms of recall, precision, and accuracy, along with excellent AUC values ranging from 0.85 to 0.89. While previous studies have achieved promising results using artificial neural network models, most have focused on imaging data, and the use of DNA methylation profiles as features remains relatively uncommon. Among the limited research using methylation data, few have attempted to classify malignant cancer stages, particularly in distinguishing early-stage disease from late-stage disease. Additionally, many models do not prioritize minimizing feature sets, which may reduce model interpretability and increase the risk of overfitting. Racial disparities in DNA methylation patterns are also often overlooked, with limited efforts to develop race-specific models despite known imbalances in publicly available datasets. Rather than attempting to learn a single model that generalizes across all racial groups, our primary objective was to build a high-performing, interpretable stage classifier within a specific race (White cohort) and then examine how its performance degrades in Asian and Black or African American patients. This design reflects a realistic scenario where models are trained in data-rich majority populations but deployed more broadly, and our findings argue for race-specific modeling and feature selection. To address these gaps, we utilized DNA methylation data and applied a combination of DMP and recursive feature elimination (RFE) to identify a minimal, high-impact set of features. We further validated the selected biomarkers using COSMIC (Catalog Of Somatic Mutations In Cancer) and cBioPortal [33] to ensure biological relevance. Our study introduced a race-aware MLP model focused on prostate cancer stage classification that specifically distinguished early stages from late stages, demonstrating the importance of both racial context and minimal feature sets in developing more accurate and equitable diagnostic tools.

## Methods

### Ethical Considerations

No human participants were directly involved in this study. All data were obtained from the publicly available Genomic Data Commons (GDC) TCGA-PRAD (The Cancer Genome Atlas Prostate Adenocarcinoma) dataset accessed via the University of California, Santa-Cruz (UCSC) Xena Browser (UCSC Xena compiles processed, deidentified The Cancer Genome Atlas [TCGA] files) [34,35]. The TCGA program collected specimens under institutional review board (IRB) oversight and with donor informed consent. Data made available through TCGA/GDC are deidentified and intended for secondary research use. As this work involved secondary analysis of publicly available, deidentified data, it did not require additional local IRB approval.

### Study Design

**Figure 1.**
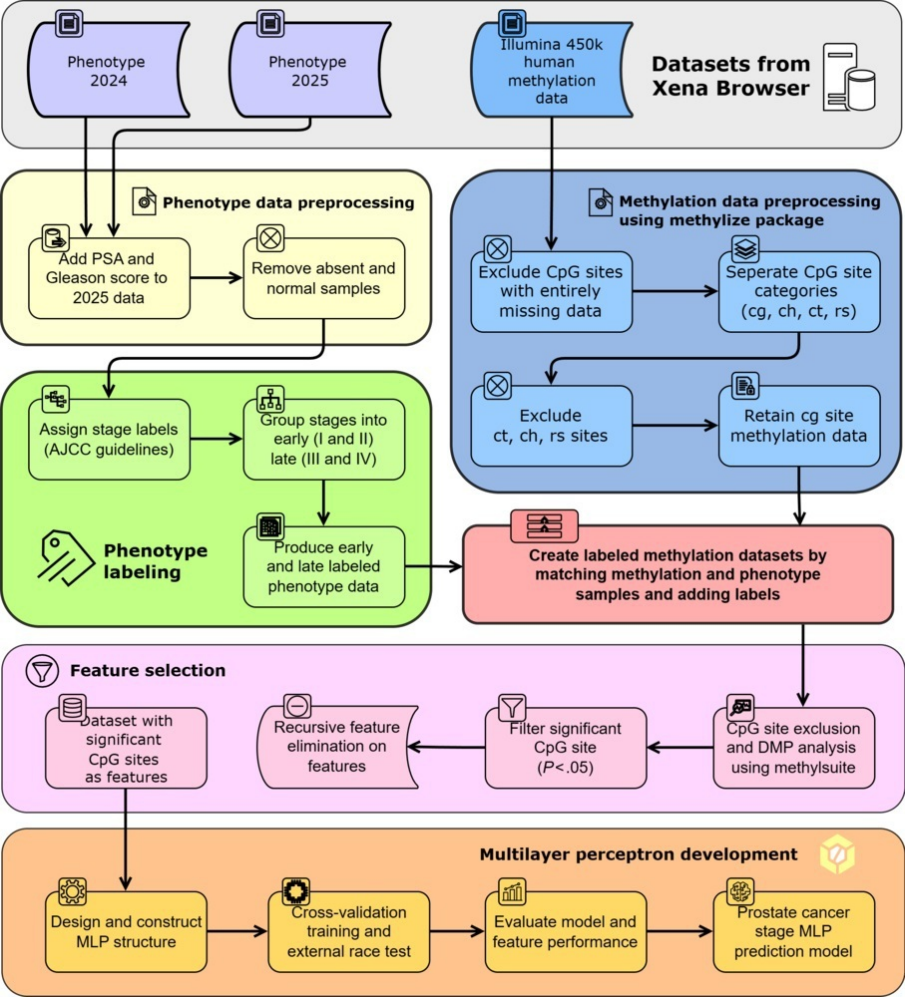
Datasets were sourced from Xena Browser and processed independently. Phenotype data were standardized for labeling, while Illumina 450k methylation data were preprocessed using the methylize package. CpG sites were categorized (cg, ch, ct, and rs); however, only cg sites were retained as others lacked sufficient methylation values. Phenotype samples were labeled according to AJCC 7th edition guidelines and binarized into “early” (stages I and II) and “late” (stages III and IV) groups. Following sample matching, feature selection was performed using methylsuite for differentially methylated position (DMP) analysis, followed by recursive feature elimination (RFE) to identify the most informative CpG sites. These features were used to develop a multilayer perceptron (MLP) model, featuring cross-validation and external race testing, to predict prostate cancer stages. AJCC: American Joint Committee on Cancer; PSA: prostate-specific antigen.

This study implemented data collection, preprocessing, feature selection, and MLP modeling and evaluation, as seen in Figure 1. These methods were conducted with Python (version 3.12.3; Python Software Foundation) programming language and the necessary libraries using Visual Studio Code editor (version 1.95.3; Microsoft Corp).

### Data Gathering and Preparation

The data used in this study were obtained from the UCSC Xena Browser, specifically from the GDC TCGA-PRAD cohort [34,35]. The data included Illumina HumanMethylation450 BeadChip data and the corresponding phenotype information, both downloaded in February 2025. However, the latest phenotype presented missing fields essential to the labeling process. To supplement missing fields in the latest phenotype release, particularly those essential for the labeling process, an earlier version of the phenotype dataset retrieved in early 2024 was also incorporated. The complete version of the phenotype dataset is available in Multimedia Appendix 1.

The Illumina 450k DNA methylation dataset consists of approximately 450,000 CpG sites, with β values ranging from 0 to 1 (indicating the methylation status: hypermethylated or hypomethylated). However, in the dataset, there are several CpG sites for which all values are null in all samples and partially null in some samples, indicating that no methylation data are available at all for those sites. In this case, not only are the null cells ignored, but also all columns of completely empty CpG sites are excluded from the feature selection process, and partially empty CpG sites are replaced with 0 to indicate that the sites are not methylated, ensuring that only features with valid information are included in the subsequent analysis.

The DNA methylation dataset consists of 4 types of CpG sites, namely, cg, ct, ch, and rs, each representing a specific location on the DNA where methylation occurs. Among the 4 types, cg-type CpG sites are the most dominant, accounting for more than 90% of the data. In contrast, ct, ch, and rs types have high levels of missing values, with ch containing more than 60% null values and rs being entirely null. Owing to the high proportion of missing values in these 3 types and in order to maintain data quality and consistency, this study only used features derived from cg-type CpG sites, while features of ct-, ch-, and rs-type CpG sites were excluded from the analysis. After obtaining the cg dataset, the samples were separated based on each race in the phenotype data. The data of the race with the highest number were used for training and testing. Each race dataset presented partially missing values in the cg data. To handle this, the mean imputation method was used to compensate for the missing values [36,37]. The remaining racial samples were used for external validation of the possibly racially based characteristics of the methylation data.

### Sample Labeling

This study began by labeling each sample in the dataset based on cancer stage. Labeling was performed using the prostate cancer labeling manual from the American Joint Committee on Cancer (AJCC) Cancer Staging Manual (Table 1). There are 5 stages of prostate cancer staging. However, in the labeling process, stages IIA and IIB were combined into a single class, namely stage II, reducing the total number of stages to 4. Labeling was carried out based on these 4 stages, which were further shortened. Stages I and II were classified as “early” (the tumor has not spread to areas other than the prostate), and stages III and IV were classified as “late” (the tumor has spread to the lymph nodes or has metastasized) [38]. Several criteria that distinguish cancer stages were referred to in the AJCC Cancer Staging Manual, including T (presence of tumor), N (spread to the lymph nodes), M (metastasis), prostate-specific antigen (PSA), and Gleason score. However, since the PSA and Gleason score criteria were missing from the Xena Browser phenotype dataset in 2025, this study compensated for the missing criteria from the Xena Browser phenotype dataset in 2024.

The distinction between “early” stages (stages I and II) and “late” stages (stages III and IV) is critically important. Early stages, including stage I (very early, often microscopic, and localized within the prostate) and stage II (cancer confined within the prostate capsule), are characterized by localized disease that has not spread to the lymph nodes or distant organs. This localization makes these stages highly manageable with treatments focused on the prostate, such as surgery or radiation, typically leading to a favorable prognosis [39]. In contrast, late stages, including stage III (cancer extending just beyond the prostate capsule) and stage IV (cancer spreading to regional lymph nodes or distant organs), represent more advanced, often systemic disease. At these stages, the spread of the cancer makes complete eradication through local treatments less likely, and systemic therapies are generally required to manage the widespread disease, resulting in a less favorable prognosis.

**Table 1.** American Joint Committee on Cancer (AJCC) instructions regarding prostate cancer stage groups [38]. AJCC guidelines stratify patients into distinct groups (I, IIA, IIB, III, and IV) based on key diagnostic parameters: T (tumor size and extent), N (regional lymph node involvement), M (distant metastasis), PSA (prostate-specific antigen) levels, and Gleason score. These parameters collectively inform the cancer’s aggressiveness and spread. However, this research follows a common rationale for consolidating stages IIA and IIB into a singular group “stage II,” which is based on the following shared characteristic: the tumor remains primarily confined within the prostate (T1 or T2) without any evidence of spread to regional lymph nodes (N0) or distant sites (M0). Despite minimal differences in tumor volume or specific T2 subclassifications between IIA and IIB, their localized nature justifies a combined classification for simplified communication and initial treatment planning.

Stage group	Characteristics
I	Localized, PSA <10, Gleason ≤6
II	Localized, higher PSA (10–20) or Gleason 7
III	Locally advanced (T3)
IV	T4, nodal involvement, or metastasis

### Feature Selection

Feature selection began with a DMP analysis of all genes against the label to find methylation values that were outliers. DMP analysis serves as an initial feature-screening step rather than as a stand-alone inferential endpoint. We therefore applied a nominal probe-wise threshold of *P*<.05 as a broad initial filter to reduce CpG sites to a reduced subset before applying RFE and downstream model-based selection. Similar 2-stage strategies, where relatively lenient *P*-value filters are combined with additional feature selection, have been proposed in high-dimensional methylation workflows to balance sensitivity and computational feasibility [40]. Methylsuite uses regression models to compare β values between early and late stages at each CpG site [41] with the methylsuite Python package using the function *methylize.diff_meth_pos.diff_meth_pos*, which accepts sample ID, β value, and label. The function returns the DMP analysis results consisting of the columns “cg pos” and “P-val.” Then, from the analysis results, a cutoff limit is given at *P*<.05. Because these DMPs were further reduced via RFE and we did not claim individual CpG sites as statistically confirmed differential methylation biomarkers in the classical sense, we opted for a more permissive threshold to avoid discarding potentially informative sites. The cg number obtained from the analysis was used as a feature for the MLP model. To minimize the number of features, the RFE algorithm was further implemented by using the support vector classifier algorithm as a classifier. The RFE process produces several subsets of data that are used to evaluate the minimum number of features, where too few features cause the model to have difficulty in distinguishing between classes. To validate the biological significance of selected CpG sites, genes related to the CpG sites were tracked using Infinium HumanMethylation450 v1.2 BeadChip product files [42].

To validate the results of feature selection, gene mutation checks were performed on all selected CpG sites. Gene mutation checking starts by taking the Illumina ID of the CpG site (example: cg00000029) and matching the Illumina ID with the gene symbols available from the Infinium HumanMethylation450 v1.2 BeadChip product files. After obtaining a list of gene symbols, gene mutations can be checked by querying an external dataset of cancer genes known to have mutations in prostate cancer cases through COSMIC, matching gene symbols in the dataset, and calculating the percentage of individual gene mutations from the number of samples that have been tested and the number of samples that have recorded mutations [43]. Further validation can be performed on cBioPortal by querying gene symbols in the Prostate Adenocarcinoma dataset (TCGA, PanCancer Atlas) to obtain more detailed information about gene mutations per prostate cancer stage in the sample dataset used in this study (TCGA) [44,45].

### MLP Development

Construction of the MLP model started with determining the scheme of the MLP to be used. We used an MLP with 2 hidden layers to balance capacity and generalization. While a single layer is a universal approximator, 2-layered networks can represent important functions much more compactly, and deeper stacks (≥3 layers) add parameters and overfitting risk on small tabular datasets without consistent gains [46]. The number of neurons in each layer is usually determined heuristically, for example, by taking the average of the number of input and output neurons or using a decreasing pattern such as 32-16-8. Activation functions, such as rectified linear unit (ReLU), are commonly used in the hidden layer to improve the ability to represent nonlinear data, while sigmoid or softmax activation functions are used in the output layer according to the type of classification. As this case involved binary classification, sigmoid activation functions were used for all output layers. The final architecture selection is generally determined through experimentation and validation to avoid overfitting and ensure optimal model performance.

After determining the MLP model scheme, model training began by separating the dataset into training and testing data. The training and testing data were obtained by separating the initial dataset. The cross-validation method was implemented in the training process to prevent potential overfitting or bias in the training data, thus expanding the generalizability of the model. Model training was performed incrementally in epochs until the process was automatically stopped by an early stopping mechanism. This mechanism stops training if there is no improvement in the model’s performance on the validation data after a certain number of epochs, thus preventing overfitting. During the training process, the model was evaluated using the Adam optimizer. All training scenarios used a consistent loss function, binary cross-entropy, in accordance with the nature of the binary classification problem.

### Performance Evaluation

Model evaluation was performed using the *classification_report* function, focusing on harmonization among the *F*_1_-score, accuracy, precision, and recall metrics. Model performance was evaluated based on the results of training and testing data to ensure reliability without overfitting or underfitting. In addition, the AUC value was also used as an additional metric to assess the model’s ability to distinguish between classes.

To enhance model interpretability in classifying prostate cancer stages into “early” and “late” stages, we used post-hoc explainable AI (XAI) methods: Shapley Additive Explanations (SHAP; version 0.48.0; Scott Lundberg) and Local Interpretable Model-Agnostic Explanations (LIME; version 0.2.0.1; Marco Tulio Ribeiro). These methods provide insights into how individual features contribute to model predictions, offering both global and local interpretability [47-49].

For SHAP, 100 random training data points were used as the background distribution for SHAP calculations. DeepExplainer was chosen based on model compatibility, and SHAP values were computed on combined data between the test and validation sets. To visualize the SHAP computation results, summarization plots were used.

LIME focused on local fidelity. To obtain a global scene of feature importance in LIME, each feature’s weight applied by the model was aggregated and compiled into a data frame, which revealed patterns of feature trends and a feature ranking based on the absolute mean of each feature weight. LimeTabularExplainer was built using the test data, feature names, and class names. LIME helped confirm the consistency of important features across individual predictions and detect any variability in the model’s decision-making at the sample level.

## Results

### Prepared Data

The data for this study consisted of 2 correlated secondary datasets. The datasets were obtained through the public databases Xena Browser GDC TCGA-PRAD DNA methylation-Illumina Human Methylation 450 and Xena Browser GDC TCGA-PRAD phenotype 2024 and 2025. The DNA methylation-Illumina Human Methylation 450 dataset contains 553 samples with TCGA barcodes and 486,428 identifiers or CpG sites with beta values (0-1), as shown in Multimedia Appendix 2.

The GDC TCGA-PRAD phenotype 2024 and 2025 datasets have 572 samples in the form of TCGA barcodes and 88 identifiers in the form of categorical clinical data, as shown in Multimedia Appendix 1.

The initial DNA methylation dataset consists of a total of 486,428 CpG site features and a total of 893 samples, and the phenotype data only involve 572 samples. This study only took samples that were in both datasets for model construction. From the methylation dataset with 553 samples, only 415 samples were taken, as this research only used White population data from both phenotype datasets by equalizing the TCGA barcodes of both datasets. After identifying null values, it was found that 64,728 CpG sites had null values in all samples (no methylation values at the sites). Such features were considered to have no analytical value and were excluded entirely from the dataset.

After the removal of completely empty CpG sites, the number of features was reduced to 421,699 or about 87% of the original data, and then, the samples were separated by race. The White race was the dominant race in the dataset, covering up to 415 out of 572 total samples, and was therefore used as the basis for the MLP model construction process. The remaining samples with other races were used for validation of the hypothesized race-based characteristics of the dataset. The races used for validation included Asian (12 samples) and Black or African American (60 samples). There were races in the phenotype datasets that were not covered by this study owing to “not reported” race values and an insufficient number of labels for prediction. This process ensured that only features with valid and analyzable methylation information were used in the DMP and RFE analysis stages.

### Labeling

Based on the labeling performed using the AJCC Cancer Staging Manual guidelines, each sample in the dataset was successfully classified into the appropriate prostate cancer stage. Some samples had missing values in one or two of the main criteria (T, N, or M), but no samples had missing values in all 5 criteria at once. To handle this, missing values in the T, N, and M categories were treated as T0, N0, and M0, respectively, which were assumed to be conditions without significant spread or growth. Meanwhile, the PSA and Gleason columns had no missing values and could be fully used for labeling. From a total of 415 White race samples, the label distribution was obtained as represented in Multimedia Appendix 3.

This distribution showed a predominance of samples at advanced stages (stage II), reflecting possible population bias or the availability of more data for advanced cases. The labeling process was performed manually and adjusted to the AJCC classification scheme to ensure accuracy and consistency between samples. All label data were then used for further analysis, including classification or clustering of prostate cancer stages.

### Feature Selection

Feature selection was performed in 2 stages, namely DMP analysis using methylsuite and advanced feature reduction using the RFE algorithm. The goal of this process was to obtain a minimum subset of CpG site features that are most relevant to prostate cancer staging.

The implementation of DMPs using methylsuite started by excluding CpG sites that had been recorded as insignificant or had no correlation to oncology from journals or public databases, which resulted in the elimination of 137,111 CpG sites [41,50-55]. The remaining 281,723 features were then subjected to DMP analysis using a *P*-value cutoff of <.05 to evaluate their influence on the label. DMP analysis with a cutoff of *P*<.05 resulted in 6501 CpG sites that were significant to the label. The cutoff of *P*<.05 was chosen as the default value because it is considered to provide a balance between selectivity and the number of features that can still be processed efficiently.

Then, the RFE algorithm was used to evaluate and select the most informative features from the 6501 CpG sites. RFE was applied by gradually generating several feature subsets, namely by reducing the number of features to 100, 90, 80, 70, and 60. Each of these feature subsets was used to form a different dataset for each race that would be used for training and testing classification models. Each dataset had its own characteristics depending on the number of features and estimators used in the RFE process. The final feature selection considered the balance between model complexity and classification performance, with the aim of avoiding the risk of overfitting or underfitting.

The biological relevance of features to labels was validated by checking several online datasets. Reference genes were searched from 100 selected CpG sites. The reference genes were obtained from the Infinium HumanMethylation450 v1.2 BeadChip manifest file by matching CpG sites to gene symbols. A total of 58 symbols were obtained from sites that had reference genes. The 58 gene symbols were matched with the COSMIC Cancer Browser tool by selecting prostate cancer tissue, exporting all data about genes that are often found in prostate cancer, and recording the number of samples and the number of mutations that occur. Genes with a mutation rate above 5% are shown in Table 2.

Based on the COSMIC database, the selected genes showed low mutation frequencies in prostate cancer samples. *MACROD2* led with a mutation rate of 28.6%, followed by *MAGI2* (24.37%), *FAM155A* (22.84%), and *DLGAP2* (22.14%). These 4 genes alone were mutated in more than one out of five cases, suggesting a potential role in the pathogenesis of prostate cancer.

While classical tumor suppressor genes, such as *TP53* and *PTEN*, are not included in this list, the mutation rates observed here may reflect less well-known but recurrent alterations in prostate cancer stages. Notably, genes, such as *FMN2*, *NTM*, and *L3MBTL4*, also showed relatively comparable mutation rates, raising questions about their functional significance. However, COSMIC does not provide context regarding cancer stage (early vs late disease), leading to a lack of information on whether these mutations occur exclusively in early- or late-stage tumors and how external factors, such as age and race, may influence these mutations. However, further validation using cBioPortal, which provides mutation data linked to cancer stages and clinical outcomes, will be crucial to determine whether these frequently mutated genes are enriched in early-stage or late-stage prostate cancer.

The same gene symbols were evaluated using TCGA data via cBioPortal, and those genes with an alteration frequency of 5% or higher were selected, resulting in the OncoPrint shown in Multimedia Appendix 4. Visual inspection of selected genes, including *DLGAP2*, *PCDH17*, *CALB1*, and *VGLL2*, revealed distinct patterns of genetic alterations across prostate cancer stages, ranging from T2A and T2C (early) to T3A and T4 (late). Notably, *DLGAP2* and *VGLL2* exhibited prominent deep deletions (represented in blue) found across various stages but predominantly in early-stage prostate cancer, suggesting a potential tumor suppressor role. In contrast, *CALB1* and *PENK* displayed consistent amplifications (represented in red), especially in late-stage samples, indicating a possible association with disease progression or aggressive traits. According to the OncoPrint results, race and age at diagnosis did not show a strong correlation with gene alterations, as their distributions appeared random and inconsistent across cancer stages. These stage-associated alteration patterns reinforce the likelihood that these genes may contribute differentially to prostate cancer. This visual stratification, when aligned with earlier COSMIC mutation rates, supports prioritizing these genes for further analysis based on mutation type, frequency, and stage-specific behavior in clinical samples.

**Table 2.** Genes with a mutation rate above 5% in prostate cancer.

Gene	Mutated samples, n	Samples tested, n	Mutation rate (%)
*MACROD2*	616	2154	28.60
*MAGI2*	539	2212	24.37
*FAM155A*	492	2154	22.84
*DLGAP2*	477	2154	22.14
*FMN2*	321	2154	14.90
*NTM*	305	2154	14.16
*L3MBTL4*	291	2154	13.51
*SHISA9*	276	2154	12.81
*PCDHGA4*	218	2154	10.12
*FBXL7*	189	2154	8.77
*GABRB3*	165	2154	7.66
*PCDH17*	127	2154	5.90

### MLP Performance

Based on conventional methods of consideration and preliminary experiments, several MLP architectures were selected to be tested on numerical data. The structures used consisted of 2 main categories, namely MLPs with 1 hidden layer and those with 2 hidden layers. For the architecture involving 1 hidden layer, the number of neurons used varied among 8, 16, and 32, with ReLU or sigmoid activation functions and dropouts between 0.2 and 0.4 to prevent overfitting. Meanwhile, the architecture involving 2 hidden layers was designed with a combination of neuron counts, such as 16‐8 and 32‐16, using consistent activation functions between layers (ReLU or sigmoid), as well as customized dropouts in each layer. The selection of these combinations aims to evaluate the effect of network depth, neuron number, activation function, and dropout on the classification performance of the model.

To identify weaknesses in model performance, we used stratified 3-fold cross-validation to maintain class balance in every split, keep approximately 110 samples per validation fold for stable estimates while reserving sufficient training data, and limit the computational burden of the nested feature-selection pipeline, which is an accepted trade-off in small, high-dimensional biomedical datasets. Prior to stratification, all features were scaled using scikit-learn’s *StandardScaler()* function to equalize the scale of values between features and speed up convergence during training.

Experiments in this study totaled 150 scenarios trained in a maximum of 100 epochs, and the number of scenarios came from a combination of 5 feature selection datasets, 3 data splitting schemes, and 10 model architectures. All scenarios were trained using an early stopping mechanism with a patience value of 10, taken from 10% of the number of epochs. Training was stopped automatically when there was no performance improvement on the validation data for a specified number of epochs. In addition, all optimizers used had a learning rate of 0.001 consistently across all training scenarios to maintain the stability of the reweighting process.

Table 3 presents the top 5 best-performing models among the 150 experiment scenarios, and Table 4 presents the top 5 best-performing models utilizing each individual feature count (ranging from 60 to 100).

**Table 3.** Top 5 best-performing models among the 150 experiment scenarios.

Features	Data split (training/testing data)	CV^a^ Acc^b^ (%)	CV AUC^c^ (%)	CV *F*_1_^d^ (%)	Test Acc (%)	Test loss (%)	Test AUC (%)	Test *F*_1_ (%)	Asian Acc (%)	Asian *F*_1_ (%)	Black Acc (%)	Black *F*_1_ (%)
90	80/20	95	99	95	98	15	100	98	25	29	43	49
60	80/20	95	98	95	93	20	98	92	33	33	42	47
100	90/10	95	98	95	95	16	99	95	8	13	47	52
70	80/20	95	99	95	95	15	100	95	8	13	48	54
100	80/20	95	99	94	99	14	100	99	8	13	47	52

a CV: cross-validation.

b Acc: accuracy.

c AUC: area under the curve.

d *F*_1_: *F*_1_-score.

**Table 4.** Top 5 best-performing models utilizing each feature count.

Features	Data split (training/testing data)	CV^a^ Acc^b^ (%)	CV AUC^c^ (%)	CV *F*_1_^d^ (%)	Test Acc (%)	Test loss (%)	Test AUC (%)	Test *F*_1_ (%)	Asian Acc (%)	Asian *F*_1_ (%)	Black Acc (%)	Black *F*_1_ (%)
60	80/20	95	98	95	93	20	98	92	33	33	42	47
60	90/10	93	98	93	95	12	100	95	17	17	43	49
60	80/20	92	96	92	94	21	99	94	17	24	43	50
60	70/30	91	95	91	93	24	98	93	25	29	32	35
60	70/30	89	95	88	94	18	99	94	25	29	40	45
70	80/20	95	99	95	95	15	100	95	8	13	48	54
70	90/10	94	98	94	98	18	100	98	8	3	38	42
70	80/20	91	94	91	86	49	83	84	25	29	52	58
70	90/10	90	96	90	95	22	100	95	0	0	33	36
70	90/10	90	95	89	100	28	100	100	8	13	42	48
80	80/20	95	99	94	92	24	98	91	25	33	42	48
80	90/10	93	98	93	95	12	99	95	8	3	33	37
80	90/10	91	96	91	98	19	100	98	0	0	38	42
80	90/10	91	97	90	86	33	95	83	33	42	58	63
80	90/10	91	96	90	76	38	99	66	83	76	83	76
90	80/20	95	99	95	98	15	100	98	25	29	43	49
90	90/10	94	98	94	100	11	100	100	8	3	35	39
90	90/10	92	97	92	98	16	100	98	0	0	35	39
90	70/30	90	94	90	98	16	100	98	8	13	38	45
90	80/20	89	96	89	93	29	97	92	25	33	43	50
100	90/10	95	98	95	95	16	99	95	8	13	47	52
100	80/20	95	99	94	99	14	100	99	8	13	47	52
100	90/10	93	96	93	98	13	99	98	0	0	37	41
100	90/10	92	98	92	100	12	100	100	8	3	37	40
100	90/10	92	98	91	76	37	95	66	83	76	83	76

a CV: cross-validation.

b Acc: accuracy.

c AUC: area under the curve.

d *F*_1_: *F*_1_-score.

Post hoc interpretability analyses using SHAP and LIME were conducted only on the best-performing MLP model, which was selected based on its superior generalization performance in the validation and test sets. This model consisted of 90 input neurons (equal to the selected feature count), 1 hidden layer with 32 neurons having a sigmoid activation function, a final sigmoid output layer, and the Adam optimizer. Regularization was applied using a dropout rate of 0.4 to mitigate overfitting.

**Figure 2.**
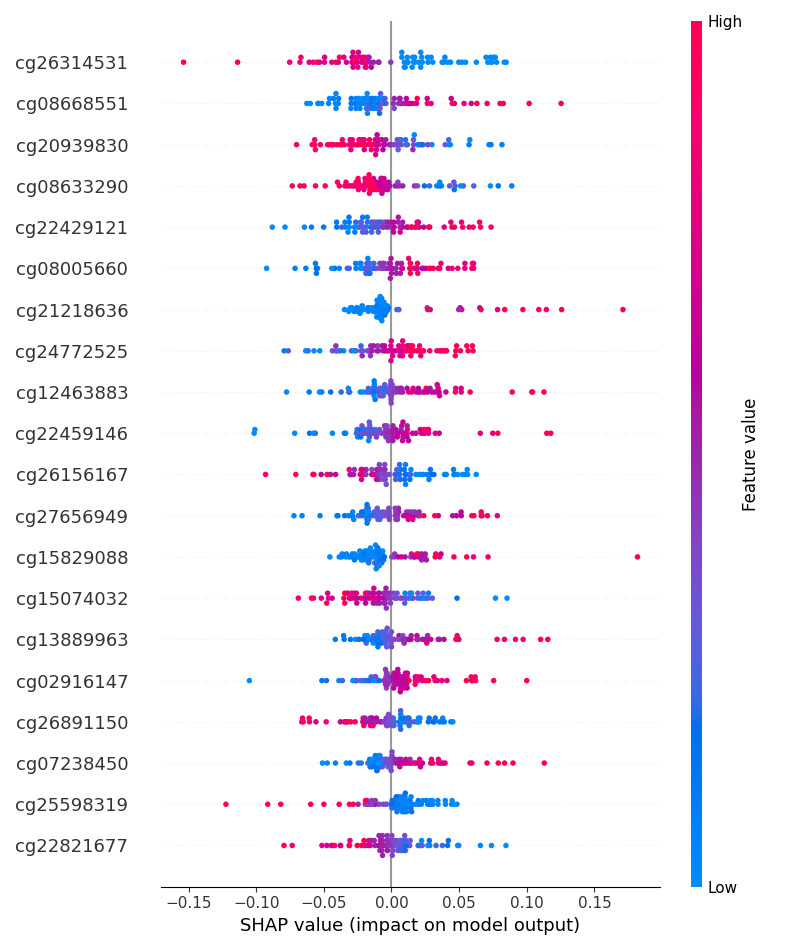
Shapley Additive Explanations (SHAP) summary plot of the top 20 most influential features. Each point represents a sample, with the x-axis showing the SHAP value (impact on model output) and the y-axis listing the top CpG sites ranked by importance. Colors represent the feature values (red=high, blue=low). Features with wider spreads and denser point distributions have a greater influence on the model’s predictions.

**Figure 3.**
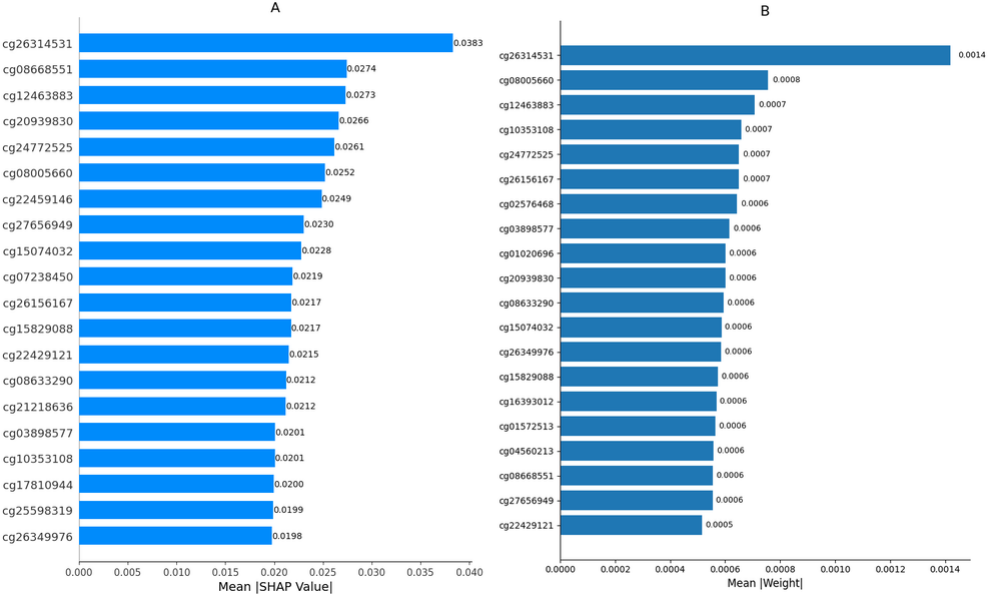
Comparison of the top 20 most influential features identified by Shapley Additive Explanations (SHAP) (A) and Local Interpretable Model-Agnostic Explanations (LIME) (B). The SHAP bar plot displays the average absolute SHAP values for each feature, indicating their overall contribution to the model’s predictions across all samples. The LIME bar plot shows the average absolute weight assigned to each feature when locally approximating the model’s output.

A total of 184 samples were used for SHAP analysis, including 100 randomly selected training samples as background distribution and 84 combined samples from the validation and test sets for explanation. LIME was applied to the same set of 84 samples to generate local explanations. Figure 2 shows the SHAP summary plot, which highlights the most influential features across the model’s predictions. Figure 3 presents a comparison of SHAP and LIME feature importance rankings.

cg26314531 was identified as the most important feature by both SHAP and LIME. Annotation analysis involving the Infinium HumanMethylation450K v1.2 product files showed that the CpG lies in a regulatory region of the genome where methylation changes are likely to influence gene activity, and this provides a biological explanation for its importance in the predictive model [56]. These findings suggest that cg26314531 may represent an epigenetic marker associated with regulatory changes relevant to cancer progression.

## Discussion

### Principal Findings

This paper proposes a race-based prostate cancer stage detection framework, which distinguishes between early and late stages, by utilizing bioinformatics feature selection (DMP) and statistical (RFE) approaches on patient DNA methylation data. This framework is designed to minimize the number of features (CpG sites) used to build a robust and accurate MLP model for classifying prostate cancer stages. By minimizing the number of features required, this approach provides several advantages for both patients and hospitals. These include reduced implementation costs due to the absence of large computational resources, as well as allowing patients to target specific CpG sites in the NGS process, so that the NGS process for irrelevant CpG sites can be eliminated. The generalization of the model is race-based owing to the fact that DNA methylation data are highly influenced by disparities in lifestyle, genetic inheritance, and habits that vary by race [57-59].

In the labeling process, the distribution of prostate cancer stages in the sample data showed that most patients were diagnosed at stage 2 (362 out of a total of 415 samples). Stage I accounted for only 21 samples, while stages III and IV accounted for 39 and 82 samples, respectively. This imbalance in distribution, particularly the high number of samples of stages II and IV compared with stage I, may indicate a delay in the early detection of prostate cancer among White individuals, with most prostate cancer cases being found at intermediate to advanced stages. Therefore, there is a need for accurate prostate cancer staging detection methods to reduce the mortality rate resulting from a delayed diagnosis or misdiagnosis among patients with prostate cancer.

To test the performance of the MLP-based classification framework, a series of experiments was conducted using data with varying feature count configurations but with variations in the proportion of training and testing data (data split) and racial distribution. The results showed that the performance of the MLP model was generally quite high, as indicated by the good accuracy, *F*_1_-score, and AUC values in the test dataset, which approached or reached the maximum value (1.00) in some configurations. However, a more in-depth analysis of the model performance by racial group revealed significant inequalities. To further validate the quality of the selected CpG sites, SHAP and LIME explainability methods were applied to analyze the contribution of each feature toward the model’s prediction. SHAP summary plots highlighted CpG sites, such as cg26314531, cg08668551, and cg20939830, as having the most substantial impact across all samples. LIME analysis confirmed the importance of many of these same features at the individual prediction level, suggesting that the selected features are not only statistically relevant but also biologically meaningful in driving classification decisions. The overlap of SHAP and LIME top features strengthens confidence in their interpretability and consistency, especially in terms of functional relevance to cancer progression. Further analysis revealed that, although the experiment focused on configuring the number of features, the same trend was observed when testing the model on a smaller number of features in a separate dataset. The cross-validation accuracy values tended to stabilize in the range of 89% to 95%, with a reduction in the number of features, indicating that an increase in the available information improves the model's ability to recognize relevant predictive patterns. However, some configurations with a limited number of features still showed high accuracy. This phenomenon suggests that the quality and biological relatedness of the selected features are as important as the quantity of features.

In the context of DNA methylation data, there may be biological connections between CpG sites that function in specific biological processes, such as the regulation of gene expression in certain tissues or races [60-62]. The selected features may represent functionally interconnected biological networks that, despite their limited number, are still informative enough to maintain model performance [63-65]. SHAP’s feature distribution plots also revealed that some CpG sites consistently contributed to increasing or decreasing model prediction depending on their methylation status, indicating that the biological signal captured is not arbitrary but directionally consistent with known methylation patterns associated with cancer stages.

Based on the model performance results, the disparity in performance between racial groups was apparent when evaluation metrics, such as accuracy and *F*_1_-score, were compared on a per-race basis. While the model achieved a high accuracy and *F*_1_-score overall, the performance for minority races, such as Asian and Black races, was much lower. For example, in one of the configurations where the overall test accuracy reached 99%, the accuracy for the Asian race was only recorded at 8%, with an *F*_1_-score of 0.13, while the Black race only achieved an accuracy of about 47% and an *F*_1_-score of 0.52. This disparity indicates that MLP models tend to only recognize patterns originating from certain racial groups, most likely the majority group, and fail to generalize the prediction results to other groups. One of the main causes of this bias is thought to be related to the imbalance in the number of samples between races. In the data used, the White race had a relatively high number of samples (63 samples), followed by the Black race (60 samples), while the Asian race had only 12 samples. This imbalance caused the model to receive more information during the training process from the majority group (White race), and thus, it was able to recognize their patterns better. In addition, the feature selection process conducted earlier was most likely based on the majority group, causing the selected features to be less relevant for minority races, and thus, the biological characteristics captured in the DNA methylation profiles of each race may produce different patterns. In the context of this study, the CpG site features selected as the basis for prostate cancer stage classification appeared to be more representative of the White group used for feature selection data and MLP model training, causing information on specific biomarkers in other races to be underrepresented. These biological differences may reflect interconnected genetic, epigenetic, environmental, and social factors that influence the methylation patterns of each race. The impact of this imbalance is not only limited to the performance of MLP models but also reaches wider areas of medical practice and public health. If predictive models are built based on data that are not racially inclusive, the risk of bias in diagnosis, prognosis, and clinical decision-making is higher, especially for racial minority groups. Therefore, the results of this study emphasize the importance of population representation aspects in DNA methylation data, as well as the need for methods that consider biological and demographic diversity in every stage of building artificial intelligence models for biomedical applications. Moreover, model interpretation tools, such as SHAP and LIME, offer valuable insights into whether feature contributions remain consistent across racial groups. In this study, these tools helped identify whether certain CpG sites unequally influenced predictions for specific races, potentially revealing biases in the model or guiding future fairness-aware feature selection strategies.

### Relation to Prior Work

Numerous studies have explored cancer classification using either DNA methylation data or deep learning techniques. However, to our knowledge, no studies have combined race-aware modeling, malignant stage classification, and biologically driven minimal feature selection in prostate cancer. Most existing work has focused on pan-cancer classification or benign versus malignant categorization without considering disease progression stages or racial variation in methylation patterns [66-69]. Several studies have utilized DNA methylation profiles for cancer detection and classification. For example, Zheng et al [70] developed a DNN-based classifier for cancer origin prediction using TCGA’s DNA methylation data from 7339 patients having 18 different cancer origins. The model used 1-way ANOVA followed by the Tukey honestly significant difference test to select significant features, resulting in 10,360 CpG sites used in the DNN. The model was then evaluated by using 10-fold cross-validation. The model achieved an almost perfect specificity score of 99.7% and a sensitivity of 92.6%. Another proposed 2-part system by Eissa et al [32] involved metaheuristic feature selection followed by a DNN for pan-cancer and malignancy status classification, which resulted in better classification in terms of recall, and similar and higher results in precision and accuracy, with excellent AUC values ranging from 0.85 to 0.89. While both studies demonstrated the potential of methylation-based classification, they did not address malignant stage classification within a single cancer type and did not consider racial disparities in methylation signatures. Moreover, their feature selection approaches focused largely on statistical or optimization-based techniques, without combining biological relevance. In contrast, our study provides a starting point for understanding racial gaps in model performance under standard training methods. Models trained mostly on data from White participants performed very well on similar White samples, but their performance dropped sharply for Asian and Black or African American samples. This shows the risk of using models that ignore racial differences in settings where DNA methylation patterns vary across populations. It also highlights the need for future research to carefully test training methods that aim to improve fairness when using methylation-based biomarkers. We adopted a bioinformatics-driven feature selection pipeline that combined DMP analysis and RFE to identify a minimal yet informative set of CpG sites. This approach enables more interpretable and race-aware modeling, addressing both the technical and biological limitations in prior work.

### Limitations

This study has several limitations that should be addressed in future research. Most notably, the model was trained on a racially homogeneous dataset, which limits its generalizability to other populations. Our findings suggest that DNA methylation patterns vary significantly across racial groups, indicating that feature selection and model training must be performed on race-specific datasets to achieve accurate and fair classification outcomes. Without this, diagnostic models risk underperforming or misclassifying in underrepresented populations. Additionally, publicly available data were used, which introduces challenges, such as missing values and potentially outdated annotations, potentially influencing model robustness. Finally, the study was limited to single-omics data. Integrating multi-omics approaches could offer a more comprehensive view of prostate cancer progression across racial subgroups. Future work should prioritize race-aware feature selection approaches, stratified training pipelines, and advanced techniques, such as domain adaptation, to enhance both performance and equity in biomarker discovery.

### Conclusions

This study presented a race-aware MLP model for prostate cancer stage classification using DNA methylation data, with enhanced feature selection through DMP analysis and RFE. Validation with external sources (COSMIC and cBioPortal) reinforced the credibility of our feature selection process, revealing important gene alteration patterns within the dataset. The results showed that the models built and tested within the White cohort achieved high performance (average testing accuracy >95%) but performed poorly when applied to Asian and African American samples (testing accuracy <50%). SHAP and LIME interpretations further confirmed the relevance of selected CpG sites and revealed how they influenced model predictions. These findings reveal clear racial disparities in DNA methylation profiles and emphasize the critical need for race-specific feature selection and modeling strategies while demonstrating the effectiveness of using a minimal feature set to capture significant biomarkers and avoid noise introduced by population heterogeneity.

## Supplementary material

10.2196/82587 Multimedia Appendix 1 Complete version of the phenotype dataset.

10.2196/82587 Multimedia Appendix 2 DNA methylation dataset matrix example.

10.2196/82587 Multimedia Appendix 3 Distribution of sample stage groups.

10.2196/82587 Multimedia Appendix 4 OncoPrint.

## Abbreviations

ADC: apparent diffusion coefficient
AJCC: American Joint Committee on Cancer
AUC: area under the curve
CNN: convolutional neural network
COSMIC: Catalog Of Somatic Mutations In Cancer
DMP: differentially methylated position
DNN: deep neural network
GDC: Genomic Data Commons
IRB: institutional review board
LIME: Local Interpretable Model-Agnostic Explanations
MLP: multilayer perceptron
MRI: magnetic resonance imaging
NGS: next-generation sequencing
PSA: prostate-specific antigen
ReLU: rectified linear unit
RFE: recursive feature elimination
SHAP: Shapley Additive Explanations
T2W: T2-weighted
TCGA: The Cancer Genome Atlas
TCGA-PRAD: The Cancer Genome Atlas Prostate Adenocarcinoma
UCSC: University of California, Santa-Cruz
