# Supplementary material for: Artificial Intelligence Design for Race-Based Prostate Cancer Stage Classification With Multilayer Perceptron: Feature Selection Optimization Approach
Source: JMIR Form Res. 2026 Apr 16;10:e82587. doi: 10.2196/82587 (PMC13086062; doi:10.2196/82587)
Supplement: Multimedia Appendix 2 [file formative-v10-e82587-s002.docx]

Multimedia Appendix 2. DNA methylation dataset matrix example.

|  | TCGA-G9-A9S7-01A | TCGA-EJ-7792-11A | TCGA-EJ-7792-01A | … | TCGA-J4-A67L-01A |
| --- | --- | --- | --- | --- | --- |
| cg00000029 | Null | 0.1888 | 0.2635 |  | 0.1521 |
| cg00000108 | 0.9691 | 0.9670 | 0.9685 |  | 0.9726 |
| … |  |  |  |  |  |
| cg-N | Methylation value  cg-N | Methylation value  cg-N | Methylation value  cg-N | … | Methylation value  cg-N |

^a^The values in the matrix are each sample’s CpG site methylation value represented in beta values ranging from 0 to 1.
