# Supplementary material for: Artificial Intelligence Design for Race-Based Prostate Cancer Stage Classification With Multilayer Perceptron: Feature Selection Optimization Approach
Source: JMIR Form Res. 2026 Apr 16;10:e82587. doi: 10.2196/82587 (PMC13086062; doi:10.2196/82587)
Supplement: Multimedia Appendix 3 [file formative-v10-e82587-s003.docx]

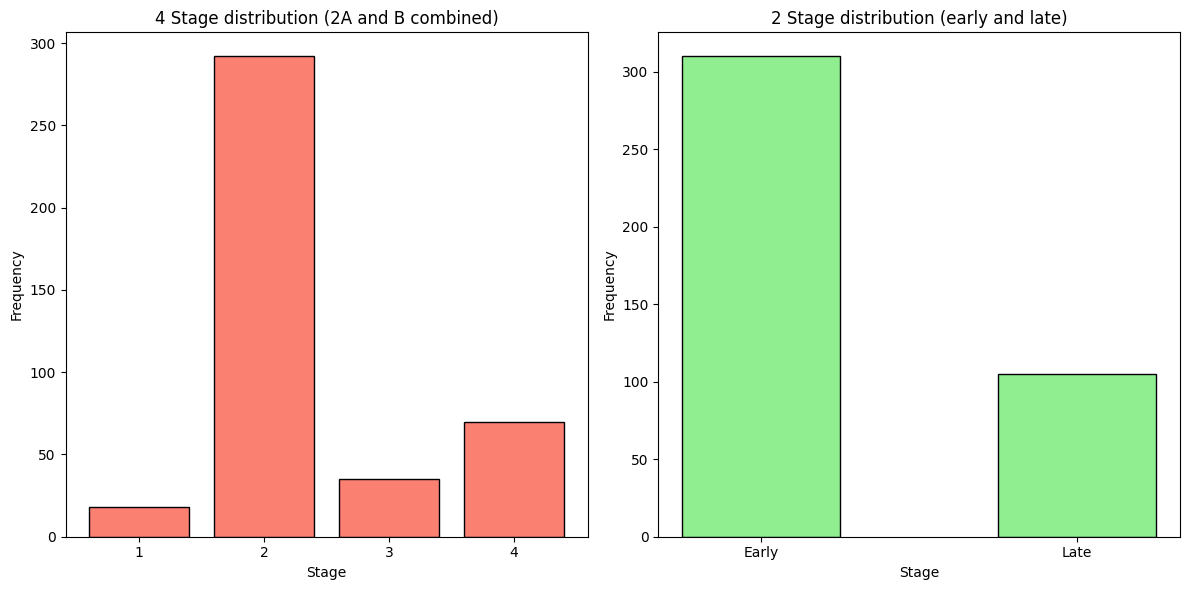


**Multimedia Appendix 3.** Distribution of sample stage groups. Stage IIA and IIB were combined to form a dominant stage II group, comprising 292 samples. In contrast, the other stage groups had significantly fewer samples: stage I had 18 samples, stage III had 35 samples, and stage IV had 70 samples. For simplification, these stages were further grouped into 2 broader categories: “early” (stages I and II; total of 310 samples) and “late” (stages III and IV; total of 105 samples).
