# Supplementary figures and images for: Artificial Intelligence Design for Race-Based Prostate Cancer Stage Classification With Multilayer Perceptron: Feature Selection Optimization Approach
Source: JMIR Form Res. 2026 Apr 16;10:e82587. doi: 10.2196/82587 (PMC13086062; doi:10.2196/82587)

| Response               | Percentage |
|------------------------|------------|
| Yes, it is a crisis    | 95%        |
| No, it is not a crisis | 5%         |

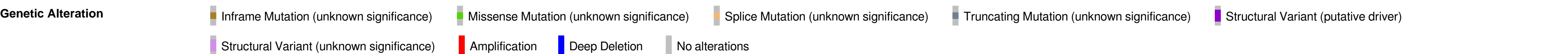

■ T2A ■ T2B ■ T2C ■ T3A ■ T3B ■ T4 — No data

Supplement: Multimedia Appendix 4 [file formative-v10-e82587-s004.pdf]
